# e-PBL with multimedia animations: a design-based research

**DOI:** 10.1186/s12909-023-04298-x

**Published:** 2023-05-16

**Authors:** Işıl İrem Budakoğlu, Özlem Coşkun, Vildan Özeke

**Affiliations:** 1 Medical Education and Informatics, Gazi University, Ankara, Turkey; 2 Curriculum and Instruction, Tokat Gaziosmanpasa University, Tokat, Turkey

**Keywords:** Medical undergraduates, Design-based research, Problem-based learning, e-PBL, Multimedia animations

## Abstract

**Background:**

This study was conducted to explore the effectiveness of online problem-based learning (e-PBL) with multimedia animation scenarios by comparing the face-to-face (f2f) PBL method with paper-based scenarios. Adapting different f2f teaching methodologies to online environments is a significant problem that urgently needs attention, particularly in health education.

**Methods:**

This study is part of design-based research and consists of three phases, which comprise design, analysis, and re-design. First, the animation-based problem scenarios were developed, and the learning environment (e-PBL) elements were organized. Then animation-based scenarios and the e-PBL environment were used, and problems related to the use of the environment were determined with an experimental study which was based on a pretest-posttest control group design. Finally, we used the following three measurement tools in the data collection process: a scale to determine the effectiveness of PBL, an attitude scale toward PBL, and the Clinical Objective Reasoning Exams (CORE). The study group in this research comprised 92 medical undergraduates (47 female and 45 male).

**Results:**

There were similar scores between the two groups (e-PBL and f2f) in terms of the effectiveness of the platforms, the attitudes of the medical undergraduates, and the CORE scores. Also, there were positive relationships between the attitude scores, grade point average (GPA), and PBL scores of the undergraduates. Another significant positive relationship was found between the CORE scores and the GPA.

**Conclusions:**

The animation-supported e-PBL environment positively effects the participants’ knowledge, skills, and attitude. Students who have high academic scores attitude positively towards e-PBL. Providing problem scenarios as multimedia animations is the innovative face of the research. They have been produced inexpensively with off-the-shelf web-based animation apps. These technological advances may democratize the production of video-based cases in the future. Although the results of this study were obtained before the pandemic, they showed no differences between e-PBL and f2f-PBL in terms of effectiveness.

**Supplementary Information:**

The online version contains supplementary material available at 10.1186/s12909-023-04298-x.

## Background

Problem-based learning (PBL), which was first used in 1969 at McMaster University in Canada [1, 2], has been used for approximately 50 years for the presentation of clinical problems in medical education [3]. PBL was first implemented in Turkey at Dokuz Eylül University Faculty of Medicine for first-, second-, and third-year undergraduate students during the 1997–1998 academic year [4].

Students are at the center of the PBL method as the “active learner” [5, 6] who are trying to understand the issue related to a given case, hypothesize about the problem, use previously acquired knowledge, define and conduct search about issues with gaps of knowledge, propose solutions, analyze the possible solutions based on the acquired information, and gain experience under the guidance of a tutor/facilitator [7]. This method can be regarded as a bridge that fills the gap between theoretical knowledge and practice [3, 8, 9]. Furthermore, the collaboration and interaction [10], as well as peer information exchange, of learners are supported by this method, especially within small groups (8–10 students) [11].

**Fig. 1:**
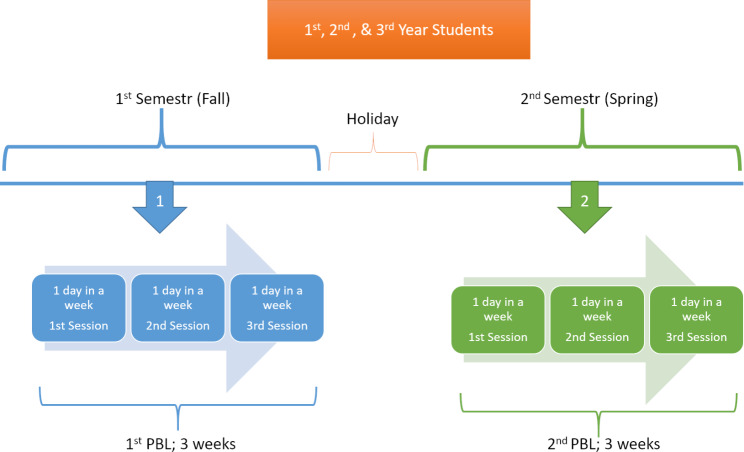
PBL application process at our faculty

In our school, we apply the PBL twice a year for the first three years (See Fig. 1). The students were divided into small groups (7–12 students), and the sessions were usually conducted as a face-to-face (f2f) interaction with a facilitator in a PBL classroom.

It is pretty challenging to organize small groups (7–12 students) and plan parallel sessions that will be appropriate for both the physical setting and facilitator need in f2f PBL sessions. On the other hand, the presentation of scenarios as hard copy-based material increases paper consumption. Furthermore, the resolution and quality of printed visual material, such as radiological/pathological images, on the scenario are restricted, and auditory or multimedia items are not commonly used. Generation Z (those born in/after 1995 – current medical students) are familiar with information and communication technologies (ICT) usage, and they prefer the Internet as a learning environment [12]. Hence, providing online learning environments in pre-medical education plays a significant role in providing physicians with 21st-century skills [13]. Online and offline resources can easily be shared via e-PBL applications between peers. It is thought that active small-group learning approaches (such as PBL) will also be successful when moved to the online environment [14, 15]. Online education is more successful and engaging if learners have the following issues; some autonomy, activities with a purpose, and interaction with peers and/or educators [16].

### Problem-based learning application on electronic media (e-PBL)

The studies [17, 18] described e-PBL as creating and sharing a case by e-mail or virtual learning environment. In the e-PBL method, students communicate and interact with each other and the facilitator through chat rooms, forums, e-mail, and interactive whiteboard applications [17, 18].

Triggering visual material and pictures, such as x-ray images and pathology results [19], in addition to the scenario, are transferred to the electronic medium via interactive videos and digital libraries [20]. The discovery of patient simulators, multimedia-supported case simulator software, and such new approaches have been reflected in PBL practice. The scenario presentation has now evolved beyond paper-based [2, 20]. For example, a study applied decision-based PBL (DB-PBL) with a virtual patient [21]. This application is a method in which the learners are together, and f2f, the facilitator is more passive, and the interaction among the students is more evident. DB-PBL allows new trials and learning to be included in the process by managing the virtual patient based on the decisions of the student group [21].

On the other hand, learners who use virtual world modalities like Second Life™ to learn with the help of fictional characters and scenarios can gain real-life experience in a safer and controlled environment [20]. Therefore, in e-PBL applications, both for sharing resources and problem-related material and for communication, synchronous (chat-MSN, WhatsApp) and asynchronous (e-mail, blog, wiki) communication media devices [20, 22] and learning management systems (Blackboard, Moodle, TopClass) that host both of them are preferred.

Instructional animations are primarily compared with static pictures in the current literature. These animations are known for their features, facilitating the perception and mental representation and helping to understand the change in space and time [23]. From this point of view, we thought it is more acceptable to categorize our e-PBL multimedia animations under “educational videos.“ Educational videos were preferred more in the multimedia presentations of the scenarios. The prominent features of these multimedia animations are quick and inexpensive production with off-the-shelf web-based animation apps.

E-learning has become indispensable during the COVID-19 pandemic [24]. During this process, it was aimed to achieve the transition to e-learning very quickly [15]. In a study that aimed to question the most successful pedagogical method for an e-learning environment, the participants and faculty members stated that video-supported PBL was considerably effective [25]. Many faculties have developed new applications that remodel the process for transferring the already existent f2f PBL sessions onto an online platform [15, 25–27]. In a study, while researchers presented their sessions using Zoom™, they used Google Docs™ as a Whiteboard application [26].

Moreover, a second facilitator can contribute to the e-PBL session as an active participant and give instant feedback to the learners instead of evaluating the f2f session process and the participants using records [26]. This was also reported to be a positive property in that study. Adapting different f2f teaching methodologies to online environments is a significant problem that urgently needs attention, particularly in health education.

In this study, we tried to make a difference in the presentation of PBL scenarios (animation based) and the sessions (online). Then we experimented with comparing the new style with the oldest one because f2f PBL has been commonly known and used for many years.

### The aim of the research

The main aim of this study was to use animation-based problem scenarios in online PBL (e-PBL) sessions and test the effectiveness of the e-PBL. The main question is; “Is e-PBL with animations an alternative to f2f PBL”? The sub-problems of this study were as follows:

1. Is the e-PBL environment (online) functional and effective?
2. How do the medical students’ attitudes differ toward PBL sessions in terms of the platform they used to join (f2f/online)?
3. Is there a relationship between the attitudes of the medical students toward the PBL sessions, their grade point averages (GPAs) (academic success), and PBL scores?
4. How do the clinical objective decision-making skills of the medical students differ in terms of the platform they used to join (f2f/online)?
5. Is there a relationship between medical students’ clinical objective decision-making skills, GPAs (academic success), and PBL scores?

## Methods

### Design

**Fig. 2:**
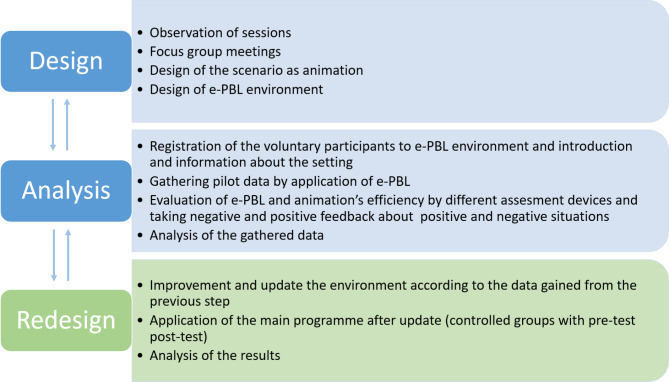
DBR plan and workflow

This study is the last phase of a project with design-based research (DBR) methodology. The design-based research method is a cyclic design-analysis-redesign model [28, 29], which took 22 months. We are sharing the project’s final results with this paper. Unless we do not mention the big picture (Fig. 2), the missing parts may blur this paper.

In the design process, which took 12 months, we made observations, discussions, and meetings about how an ideal PBL environment should be presented online. Then we designed and developed animation-based problem scenarios and organized the e-PBL learning environment.

In the analysis section, which took four months, we educated the volunteer users about the e-PBL setting. We gathered data using animation-based scenarios in this setting and analyzed the data. The users’ (participants and tutors) feedback allowed us to change the issues related to animations and the e-PBL setting. We gathered different data types and analyzed them to get a convenient e-PBL setting with animations.

We implemented the changes thoroughly and smoothly during the re-design process, which took six months. Then we evaluated the effectiveness of the e-PBL setting with an experimental study (pretest-posttest control group design). This paper presents the results of this experimental phase.

### Participants

The study group consisted of Gazi University Faculty of Medicine second and third-year students and faculty members managing “problem-based learning” sessions. The PBL secretariat managed assignments of students to small groups. The researchers did not make any assignments. Ninety-two second and third-year students (nine small groups) volunteered to participate in the experimental study. The volunteer students were grouped by their access to the Internet as e-PBL or f2f.

While determining the study group, we announced Whatsapp groups of students by contacting their group leader. They asked their peers who would like to join our study. After students became volunteers, we asked them about their Internet access status. We selected the e-PBL groups from these small volunteer groups with unlimited Internet access. Other volunteer students without Internet or limited access were assigned to the f2f PBL group of the study. The remaining students did not agree to participate and attended f2f PBL sessions as usual.

Of the students in nine small groups, 47 were female, and 45 were male. They ranged in age between 19 and 30 years (mean: 20.57 ± 1.94). The first-year students were not included in the study group as they did not have enough experience with PBL sessions [8]. The number of participants in each small group is given in Table 1. However, we reached more volunteer students under the f2f group (N = 60) for the fourth and fifth research questions related to CORE performances.

**Table 1:** Administration schedule of PBL sessions of the study group

Scenario Board: Name	Academic Year	Group ID (N)	Platform	Session 1 Date & Time	Session 2 Date & Time	Session 3 Date & Time
Endocrinology: Why is this saddle narrow?	2nd Year	G1 (9)	e-pbl	March 25 10:58 − 12:58	March 27 11:05–12:05	April 3 10:55 − 11:55
		G2 (11)	e-pbl	March 25 12:52 − 14:42	March 28 17:51 − 19:51	April 1 17:57 − 19:37
		G3 (10)	e-pbl	March 27 20:52 − 21:52	March 29 15:59 − 16:59	April 2 16:20 − 18:20
		G4 (10)	e-pbl	March 22 14:58 − 16:13	March 27 09:37 − 12:07	April 3 13:56 − 15:26
		G5 (10)	f2f	March 25 15:13–16:13	March 28 15:56 − 16:56	April 2 15:58 − 16:58
		G6 (10)	f2f	March 22 10:15 − 11:30	March 27 10:30 − 11:58	April 2 10:30 − 11:30
Gastroenterology and Digestive System: I’m not broken I’m standing	3rd Year	G7 (10)	e-pbl	April 9 19:25 − 14:00	April 15 19:16–20:56	April 17 17:59 − 18:59
		G8 (10)	e-pbl	April 9 13:43 − 15:43	April 16 14:30 − 15:30	April 18 18:12–19:12
		G9 (12)	f2f	April 3 14:30 − 16:00	April 10 14:44 − 16:24	April 17 14:12–15:20

### Design of the animation-supported scenarios and online PBL setting/medium

**Fig. 3:**
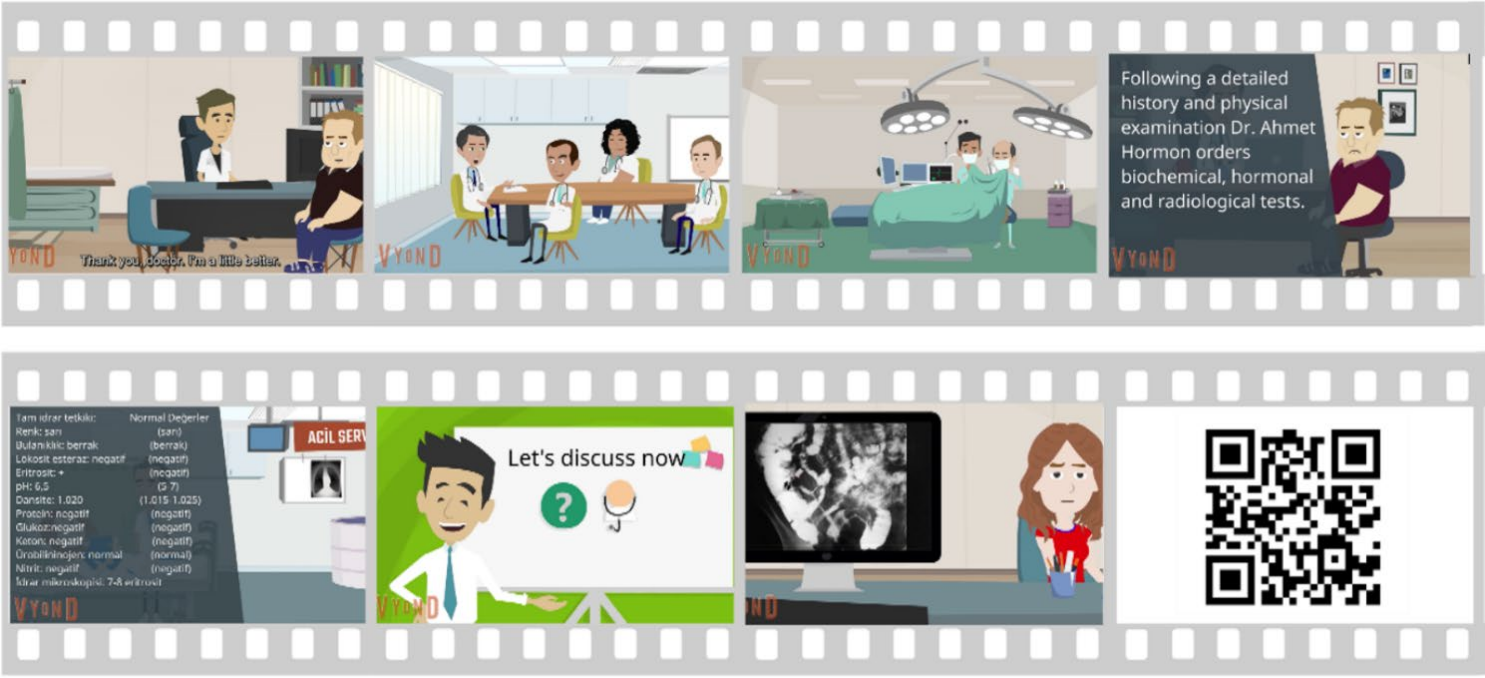
Some of the scenes of the scenarios (Please scan the QR code to watch a sample)

Thirty written and text-based scenarios from 14 systems (e.g., Endocrinology, Nervous System, Respiratory System, Cardiovascular System, Gastrointestinal System, etc.) were colored and revised using visual processing software. Vyond.com™ web applications were used to design and develop the animations. Whenever the characters in the scene needed to exhibit disease symptoms (e.g., skin rash, facial wound, spots), visual processing software was used again. Some of the scenes of the scenarios transformed into animations are presented in Fig. 3. Additionally, scenario examples in Turkish or English are accessible at: https://youtu.be/aHjml-43bHs. The video link is also generated with the QR code in Fig. 3.

The Soundbible.com™ was used for sound effects. Volunteer students and simulated patients of our medical school vocalized the scenarios. These doublings were recorded on a cell phone or voice recorder. The participants gave written informed consent before their voices were recorded. The sounds were added to the animation scenes after reduction and processing using Audacity™ and conversion to the appropriate format. The animations were archived in video format using the .mp4 extension.

The Advancity Learning Management System (ALMS) (Istanbul, Turkey) and Perculus (Istanbul, Turkey) software systems were used for the online e-PBL medium. ALMS, developed by Advancity, is a domestic product used for the communication and posting needs of faculty members and students in formal and distance learning. Perculus provided virtual classroom applications for live/synchronous distance education. We used Perculus while managing e-PBL sessions in six e-PBL groups. ALMS was used for providing the access links for uploading learning objectives, participating URLs for the live sessions, and reviewing the previously recorded live sessions for the group participating in the e-PBL process.

On the other hand, f2f PBL sessions were conducted in small group rooms in the faculty. The facilitator and the students were all together in the same place. They used paper-based scenarios, and all the discussions were conducted physically.

### Procedures

In this study phase, data were collected with two scenarios in Spring Semester (Table 1). The e-pbl group took the scenarios with animations online, yet the f2f group took paper-based scenarios in a physical room. During the online session, the e-pbl group engaged with the facilitator and peers via chatbox, whiteboard, and talking in a live classroom. In addition, they provided additional materials (e.g. additional sources, learning objectives, etc.) asynchronously on the learning management system.

### Data Collection

#### Evaluation of PBL Effectiveness Scale

This scale is a self-report and Likert (5 points). It has two parts, one for students and the other for facilitators [30]. We used the questionnaire for the students, which consists of 16 items in three subscales; knowledge (4 items), skill (5 items), and attitude (7 items). The Cronbach alpha reliability value is 0.72 (0.86 for this research). We calculated the Chronbach alpha reliability values for each subscale as follows; 0.83 for knowledge, 0.94 for skill, and 0.78 for attitude. The whole items of the scale are given in Table 2.

**Table 2:** Evaluation of the Effectiveness of Platforms

Items	e-PBL (N = 57)				f2f (N = 32)	
	Pretest		Posttest		Posttest	
	M	SD	M	SD	M	SD
1. It would take a SHORTER SPAN of TIME if I had PBL subjects-lectures in classical education	2.95	1.16	2.96	1.12	3.13	1.13
2. I would learn BETTER if I had PBL subjects-lectures in classical education	3.18	1.05	3.39	1.10	3.78	1.01
3. I believe I am more successful in EXAMINATIONS on the lectures I learn with PBL	3.39	1.25	3.72	0.92	3.88	0.94
4. PBL effects my motivation MORE POSITIVELY compared to classical education	3.63	1.10	3.86	0.85	4.19	0.82
5. PBL provided me to associate basic sciences such as anatomy, and physiology with clinical sciences	4.00	0.87	4.18	0.83	4.28	1.02
6. PBL improved my ability to describe and communicate about the subjects I have learned to others	3.84	1.03	3.88	0.98	3.94	1.01
7. I think PBL will help my lifetime learning	3.72	1.05	3.84	0.96	3.78	1.07
8. PBL helped me learn by myself by searching	3.84	0.92	3.89	0.99	3.91	0.93
9. PBL contributed to me use the Internet more to reach the information	3.93	1.00	3.93	0.94	3.91	0.82
10. PBL contributed me to work more than ever before	2.96	1.16	3.19	1.23	3.06	1.22
11. Group work in PBL contributed to my learning process	3.82	0.89	3.95	0.95	4.03	0.86
I believe PBL polished my skills on the issues below and prepared me for professional life.						
12. Reasoning skills	4.12	0.80	4.07	0.65	4.28	0.81
13. Problem-solving skills	4.12	0.87	4.16	0.65	4.31	0.69
14. Judgement skills	4.00	0.89	4.07	0.70	4.09	0.73
15. Skills to approach patient biopsychosocial as a whole	4.00	0.89	4.14	0.67	3.91	1.06
16. Communication skills	3.79	1.01	3.98	0.83	3.94	0.91
**General Mean**	**3.71**	I agree	**3.83**	I agree	**3.90**	I agree

#### Attitude toward PBL Scale

The scale is a self-report and Likert type (5 points). It consists of 38 items in six subscales. The Cronbach alpha reliability value for the original scale is 0.86 (0.95 for this research).

We calculated the Chronbach alpha reliability values for each subscale as follows; problem-solving (0.95 for seven items), group work (0.87 for ten items), self-directed learning (0.79 for six items), web platform (0.68 for six items), the scope of the course (0.76 for five items), and facilitator (0.65 for four items) [31]. A sample item for each subscale is provided as respectively; “I can generate various hypotheses for solving problems,“ “I would like to work with the group to solve the problem,“ and “I prefer to achieve goals myself, rather than the facilitator informs me,“ “I do not like to learn from lecture notes and reading passages on the web,“ “The topics I learned in this scenario are useful for my medical education,“ and “The facilitator did not ensure the active participation of all group members.“

#### Clinical reasoning and decision-making skills examination

Clinical judgment skills are specific to the scenario and the case and may differ from one case to another [2, 32]. The Clinical Objective Reasoning Exam (CORE) evaluates these skills [33] and asks questions related to a relevant problem scenario. Among the options, it rates the things that need to be done, the situations that do not matter whether it is done or not, and the situations that are harmful if done, with positive and negative points. The score may increase or decrease according to the situation of benefit or loss; the determinant is the experts in the field and the person or people who produced the scenario. The difference between the CORE exam and the achievement test is that no single correct answer for each question and bipolar assessment can be considered. We used scenarios from Endocrine System for the 2nd year and the Gastrointestinal and Digestive System for the 3rd year.

We gathered GPA scores until the beginning of the study of the students. The CORE and PBL scores did not contribute to the GPA scores.

### Data collection process

The data collection process comprised a pretest and posttest for the e-PBL group, whereas only the posttest was used for the f2f PBL group. Because CORE is a problem or case-specific posttest, it was applied to both groups at the end of the scenario. The data collection process can be viewed in Table 3.

**Table 3:** Application of the data collection devices on the groups

	Pretest		Posttest		CORE
	Evaluation of PBL effectiveness	Attitude	Evaluation of PBL effectiveness	Attitude	
f2f	-	-	√	√	√
e-PBL	√	√	√	√	√

### Data analysis

We conducted the statistical analysis using SPSS v.22.0 for Windows (Chicago, IL, USA). Categorical variables were presented as frequencies and percentages, whereas continuous variables were reported as means ± standard deviations (SD). The chi-square or Fisher’s exact test was used to compare categorical variables. A t-test was used to compare continuous variables. In addition, the Pearson multiplication of the momentum correlation coefficient was used. All tests were two-tailed, and a p-value of < 0.05 was considered to indicate statistical significance.

### Ethical considerations

Participation was voluntary, and informed consent was obtained from all subjects. All methods were carried out following relevant guidelines and regulations. Çukurova University Institutional Review Board approved the study (code:2016-59).

## Results

### Effectiveness of e-PBL environment (online)

Fifty-seven participants of the e-PBL and 32 participants of f2f PBL fulfilled the data collection devices completely. According to the results, the animation-supported e-PBL environment positively effects the participants’ knowledge, skills, and attitude. On the other side, the thoughts of the f2f PBL learners about the effectiveness of PBL were positive, and the scores were high. The independent samples t-test showed no statistically significant difference between the posttest results of e-PBL and f2f PBL groups regarding process efficacy (t _87_ = 0.538; p > .05).

**Fig. 4:**
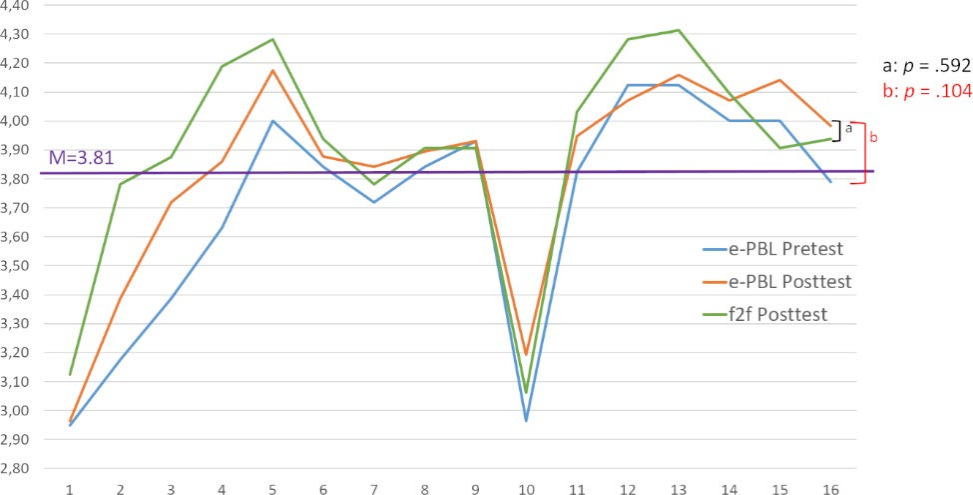
Evaluation of participants for animation-supported e-PBL effectiveness

Furthermore, paired-sample t-test results indicated that there was no significant difference between the pretest scores of the previous conventional PBL educated group and posttest results after e-PBL sessions for the animation-supported e-PBL group (t_56_ = 1.655; p > .05). Although the e-PBL group had higher scores when compared to the f2f group for some items (items 7, 9, 10, 15, and 16), there was no significant difference between the platforms (Fig. 4).

### Attitudes of the medical students toward e-PBL sessions

**Fig. 5:**
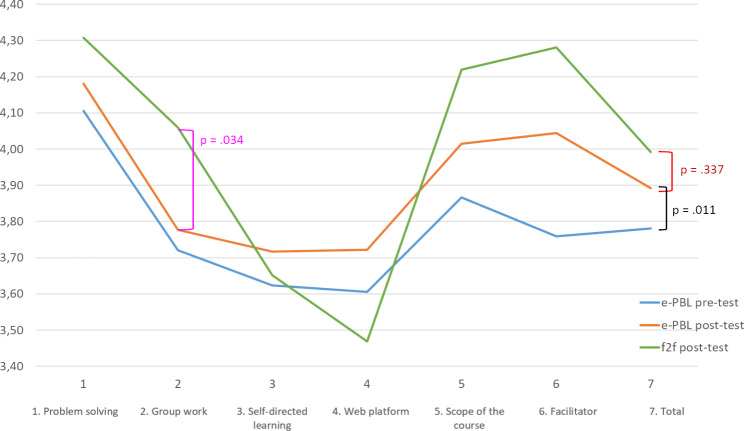
Change in the attitude scores of the participants toward PBL

The attitude scores of participants toward the animation-supported e-PBL are presented in Fig. 5. Although an evaluation of the scores revealed that their attitudes were positive (at the level of “I agree”), there was no statistically significant difference between the scores of the f2f (3.99 ± 0.41) and e-PBL (3.89 ± 0.49) groups according to independent samples t-test results (t _87_ = 0.965; P > .05). Only in the sub-dimension of “group work” were the scores in the f2f PBL group (4.06 ± 0.47) found to be significantly higher than those in the e-PBL group (3.78 ± 0.65) (t _87_ = 2.155; P < .05).

There was a parallelism between the pretest (3.78 ± 0.54) and posttest (3.89 ± 0.49) attitude scores of the e-PBL group, and the paired samples t-test results showed that participants had significantly higher scores in the posttest than in the pretest (t_56_ = 2.637; p < .05).

### Relationship between the attitudes of the medical students toward PBL sessions, their GPA, and the PBL scores

There were positive, statistically significant relationships between the attitude scores, GPA, and PBL scores (attitude-GPA: r = .231; p < .05), (attitude-PBL: r = .211; p < .05), (PBL-GPA: r = .247; p < .05) (Table 4).

**Table 4:** Relationship between the PBL Attitudes and CORE scores of the participants with the PBL scores and general success (intercorrelations)

Attitude Scores, Academic Achievement, and PBL Scores (N = 91; e-PBL: 60, f2f: 31)					
	Mean	SD	Attitude	GPA	PBL
Attitude score	78.04^**^	9.61			
Academic achievement score (GPA)	75.22	7.17	0.231^*^		
PBL Score	94.89	8.25	0.211^*^	0.247^*^	
**CORE Scores, Academic Achievement, and PBL Scores (N = 117**; e-PBL: 57, f2f:60**)**					
	**Mean**	**SD**	**CORE**	**GPA**	**PBL**
CORE score	57.77	18.72			
Academic achievement score (GPA)	77.39	7.33	0.188^*^		
PBL Score	95.60	7.04	0.059	0.045	

^*^ p < .05

^**^ The attitude scores were calculated out of 100 points.

### Clinical objective decision-making skills

Of the participants, 57 from the e-PBL group and 60 from the f2f group took the CORE exam. There was no significant difference in the CORE scores between the online (57.54 ± 20.12) and f2f (57.98 ± 17.44) groups (t _115_ = 0.127; p > .05) (Table 4).

### The relationship between the CORE scores, GPA, and PBL scores

There was a weak and positive correlation between the CORE scores of the participants and their academic achievement (r = .188; p < .05). There was no significant relationship between PBL scores and CORE and academic achievement.

## Discussion

In this study, we aimed to use animation-based problem scenarios in online PBL (e-PBL) sessions and to test the effectiveness of the e-PBL. While specific educational activities were continuing by f2f in 2018–2019, when the research was conducted, all of the activities were transferred to and carried out in online environments due to the COVID-19 pandemic. The findings obtained in this research showed that e-PBL conducted in an animation-based online environment was as effective as f2f PBL. This section is organized under three captions, effectiveness (RQ1), attitudes (RQ2 & RQ3), and CORE performances (RQ4 & RQ5), by merging the research questions (RQ) of the study.

### Effectiveness of e-PBL environment (online)

There was no significant difference between the environments regarding the effectiveness of the animation-supported e-PBL environment of the participants and the f2f PBL environment. In a study, the participation, communication, preparation, critical thinking, and group skills competencies of remote PBL participants were found to be significantly lower than the f2f PBL participants [24]. In another study [34], a scenario was presented online to 73 students who had taken case-based f2f lectures four times previously, and the participants implied that this application served as a more flexible working opportunity, and they did not spend money or time for transportation to school; however, they had technical problems with the camera and microphone, and discussion was more challenging when compared to a f2f environment. Students love f2f small group practice, enabling them to get together with friends and faculty more closely [14, 15, 35]. Although they are together online, they may not be able to capture the physical presence (feeling of being there) that they feel in a f2f environment [36]. The fact that the cameras are not always on [15], the microphone is activated only for those who speak, the situations of those who prefer to communicate by writing are in proportion to their writing speed, and the same opinion is no longer repeated by others, since their faster peers are ahead of them, are situations that can negatively affect the feelings of participation in an online environment. Compared to f2f PBL sessions, e-PBL lasts longer and allows everyone to speak, while others are distracted and less questioned due to their “passive participation.“ These kinds of adverse situations have been reported in the literature [26]. The data in the current study were collected just before the pandemic began, during a f2f training period. However, considering the pandemic conditions that currently exist, it is possible to say that the e-PBL process is a good alternative within the framework of the findings. In this period, when a f2f meeting is not possible, the animation-supported e-PBL application can be put into use, and the opinions of students and faculty members about its effectiveness can also be investigated.

### Attitudes of the medical students toward the e-PBL sessions

The scores of the animation-supported e-PBL group are partially lower than the attitude scores of the f2f group. However, this increase is hopeful, considering that such a change in attitude is time-dependent and will not occur quickly. Furthermore, in the pilot implementation phase of this DBR, it was seen that the students who attended the e-PBL sessions had positive attitudes after the application and thought that the e-PBL was effective [37].

Depending on the presentation style of the scenarios (paper-based (hard copy) or digital), the presentation time, training, cost, and attractiveness of the lessons varied [38]. Although there were technical and infrastructural problems during the e-PBL sessions during the live lessons, the views of the participants and their feedback on the animations revealed the importance of this study. The learners stated their satisfaction and the benefits of the animations in the other phases of this DBR through their views on open-ended items [37]. A study reported that 73% of the students in their study were more satisfied with digital scenarios and stated that they saved 90% of their time when they used digital scenarios [38]. Their research showed that videos were preferred more in the multimedia presentations of the scenarios [39, 40]. Video recordings show posture and movements (body language, gestures, facial expressions, etc.) more effectively than text-based scenario presentations [41]. The use of multimedia elements in PBL scenarios, although providing a solution to the problem of paper consumption in the f2f PBL process [8], ensures that the scenario is close to reality and also makes it easier for the learner to become more intertwined with the script when compared to its presentation in paper and pencil format [39, 40]. For example, a study stated that e-PBL programs succeeded in their video-based problem scenario presentations because the patients looked more realistic and holistic [42]. They also emphasized that these videos could be used in the traditional PBL method [42].

On the other hand, another study stated that when they switched from text-based presentations to video-based problem scenario presentations in the PBL method, they expected that the learners, who were defined as “digital natives,“ would be more interested in the videos. However, they stated that the learners preferred text-based presentations [43]. In the same study, the opinions of PBL facilitators/tutors were also taken, and they stated that there was no difference between the two methods (text/video-based) in terms of effectiveness, group discussions, dynamism, and communication skills. They found the video-based presentations to be more interesting. Moreover, they suggested that video-based scenarios should be used more selectively in problem types in determining learning objectives [43]. Lou et al. (2010) stated that the content in online learning environments should be interesting, such as animation or educational games, to attract learners’ attention and increase their motivation [20]. A positive and significant relationship existed between their attitudes toward PBL practices and their general academic achievements and PBL scores. As their attitudes toward PBL increased, their PBL scores also increased. The PBL method strongly effects learning and achievement [44, 45]. According to a study, discussing f2f in groups and collaborative working is the most critical process of PBL and learners need to develop different perspectives [46].

### Clinical objective decision-making skills

There was no significant difference between the online and f2f groups regarding the CORE performances. In addition, researchers found no difference between digital and paper-pencil-based scenario presentations regarding their efficacy and contribution to clinical reasoning skills [38]. Therefore, those who take PBL in an online environment have similar learning outcomes as those who take PBL in a f2f environment, and these two different environments are equivalent. In another study, online lectures and PBL sessions were applied to third-year medical students who could not undertake general surgery internships during COVID-19 [27]. The success of the online learners in the National Board Examination and oral exams was close to the success of the internship group in the same exams in the previous year, and no significant difference was found between them [27]. However, the researchers found that although they observed many surgical and technical skills (e.g., suturing, knot tying, nasogastric tube placement, and urinary catheter insertion), they needed a chance to apply them. Therefore, after COVID, online applications should be added to f2f training to allow the applications to be more efficacious [27].

There was a significant positive correlation between the CORE scores and academic achievement. The PBL method promotes self-learning and deep learning rather than rote learning while supporting clinical reasoning, team skills, and metacognitive awareness [47]. However, no significant relationship was found between the CORE and PBL scores.

There was also no significant relationship between the PBL scores and academic achievement. A study reported the findings they obtained based on the data of studies that they examined about the effectiveness of digital PBL (DPBL) compared to traditional PBL. It revealed that DPBL was as effective as f2f PBL in terms of knowledge acquisition [48]. Furthermore, they observed that the participants using the DPBL, which was entirely remotely run, achieved better success scores than those using the traditional PBL. In addition, they emphasized that there have been very few studies on PBL practices through distance education for educating healthcare staff (physicians, nurses, dentists, etc.), and they could find limited evidence on this issue. For example, the number of studies on the outputs, costs, and undesirable/adverse effects of DPBL in terms of skills, attitude, and satisfaction needs to be increased [48].

One of the limitations of the current study was that the study group was relatively small and the research time was short. Due to their nature, PBL sessions are planned once and last for three weeks in a period, and since the duration cannot be changed, it may be more appropriate to expand the group. In addition, with the pandemic process, it will be possible to share animation-based e-PBL sessions with more student groups.

In conclusion, the technological familiarity of faculty members and students with the e-learning environment is believed to effect their views and attitudes toward e-PBL. On the other hand, the facilitator effect can be considered a “confounding factor” in PBL sessions, either f2f or e-PBLs. PBL facilitators are faculty members with education and a certificate in this subject. In routine practice, students (one person from each group considering the group’s views) fill out the facilitator guiding evaluation form. However, it is a process with few sanctions. In e-PBL, technical skill is also a factor in addition to the pedagogical skills of the facilitator. Thus far, multimedia animations have been produced inexpensively with off-the-shelf web-based animation apps. These technological advances may democratize the production of video-based cases in the future.

## Electronic supplementary material

Below is the link to the electronic supplementary material.

Supplementary Material 1

Supplementary Material 2

Supplementary Material 3

Supplementary Material 4

## Data Availability

All data generated or analyzed during this study are included in this published article [and its supplementary information files].
